# Factors associated with increased radiation exposure in the fixation of proximal femoral fractures

**DOI:** 10.1308/rcsann.2023.0092

**Published:** 2024-04-02

**Authors:** A Elbahi, O Thomas, M Dungey, C Randall, DK Menon

**Affiliations:** ^1^ Dartford and Gravesham NHS Trust, UK; ^2^ Kettering General Hospital NHS Foundation Trust, UK; ^3^ University Hospitals of Leicester NHS Trust, UK

**Keywords:** Proximal femur fracture, Radiation, Fluoroscopy, Reduction, Fixation

## Abstract

**Introduction:**

When using radiation intraoperatively, a surgeon should aim to keep the radiation dose as low as is reasonably achievable to obtain the therapeutic goal. We aimed to investigate factors associated with increased radiation exposure in fixation of proximal femur fractures.

**Methods:**

We assessed 369 neck of femur fractures over a 1-year period in a district general hospital. All hip fracture subtypes that had undergone surgical fixation were included. We assessed the relationship between type of fracture, implants used and surgeon level of experience with the dose–area product (DAP; cGy/cm^2^) and screening time (dS). We also looked at the quality of reduction and fixation and its effect on the radiation exposure.

**Results:**

A total of 184 patients were included in our analysis; 185 patients who were treated with hip arthroplasty were excluded. There was a significant association between higher DAP and fracture subtype (*p *= 0.001), fracture complexity (*p *< 0.001), if an additional implant was used (*p *= 0.001), if fixation was satisfactory (*p *= 0.002) and operative time (*p *< 0.001). DAP was higher with a proximal femoral nail than with a dynamic hip screw, especially when a long nail was used. There was some evidence of an association between the surgeon’s level of experience and DAP exposure, although this was not statistically significant (*p *= 0.069).

**Conclusions:**

Increased radiation in proximal femur fractures is seen in the fixation of complex fractures, some subtypes, with certain types of implants used and if an additional implant was required. Surgeon seniority did not result in less radiation exposure, which is in contrast to other published studies.

## Introduction

Fluoroscopy is commonly used in orthopaedic procedures and can facilitate a decrease in operative time and improved accuracy in implant placement.^1^ However, the unquestionable benefits of fluoroscopy use must be counterbalanced by the increased risk of radiation exposure to the patient, theatre team and surgeons.

The harmful effects of radiation have long been identified and include blood-borne cancers, thyroid disease and solid organ tumours. Mastrangelo *et al* found a significantly increased risk of cancer in orthopaedic surgeons compared with radiation-exposed personnel from other specialties and unexposed workers. They found that 29% of the exposed orthopaedic group developed cancer over a 24-year period.^2^ Brain cancer was found to affect 31 physicians in Roguin *et al*’s report who were working for a prolonged period (mean 23.5 ± 5.9 years) in interventional practice using ionisation radiation.^3^ Therefore, monitoring radiation and regular audits are recommended to avoid excessive exposure.^4^ Dose–area product (DAP) and screening time (minutes:seconds, mm:ss) are used to identify radiation exposure. DAP is a measurement of the amount of energy delivered by the X-ray beam to the patient and is measured using a meter present in the fluoroscopy machine. DAP is calculated by multiplying the radiation dose delivered in the centre of the beam by the field size or area and is measured in units of cGy/cm^2^.^5^ The International Commission on Radiological Protection (ICRP) conducts regular reviews and produces guidance on radiological protection, and suggests using the “as low as reasonably achievable” principle, on the presumption that all radiation has the potential to cause harm. This concept was first introduced in ICRP Publication 93 “Managing patient dose in digital radiology”.^6^

Proximal femoral fractures can be fixed using different implants, and the most used are dynamic hip screws (DHS), cannulated cancellous screws and cephalomedullary nails. These procedures use fluoroscopy to ensure satisfactory reduction and device implantation.

The purpose of this study was to identify factors that lead to increased radiation exposure during fixation of proximal femoral fractures.

## Methods

Between March 2019 and April 2020, trauma cases with proximal femur fractures were reviewed. Of a total of 369 hip fractures, 184 needed surgical fixation and were included for analysis. Using a standardised proforma on an Excel spreadsheet, we used electronic operation note records, the Operating Room Management Information System (ORMIS), and Bluespier (Droitwich, UK) to record the procedure type and duration, implants used and surgeon grade. We classified surgeons into three categories based on their level of surgical exposure: junior surgeons were core surgical trainees and first- and second-year higher speciality trainees or equivalent; more senior registrars (years 3, 4 and 5 in training) were considered as middle-grade surgeons; senior registrars (last year of training), associate specialist doctors and consultants were categorised as the most senior surgeons. A dose report of each case on electronic radiograph visualisation software (Picture Archiving and Communication System; PACS) was used to collect data for DAP and screening time (dS) taken intraoperatively. All data were collected retrospectively.

There are various classification systems for proximal femur fractures, which can be categorised as intracapsular, pertrochanteric and subtrochanteric based on the anatomical location. As per Arbeitsgemeinschaft für Osteosynthesefragen/Orthopaedic Trauma Association (AO/OTA) classification, a pertrochanteric fracture can be simple (31A1), multi-fragmentary (31A2) or inter-trochanteric (31A3) (often called reverse oblique).^7^ Owing to a larger number of fragments in types 31A2 and 31A3 and the difficult reduction, we categorised these hip fractures as complex. Simple pertrochanteric fractures and intracapsular neck of femur fractures came under the category of a simple fracture pattern. Hips with an intracapsular fracture of the neck of the femur were considered simple pattern because they warranted internal fixation rather than hip arthroplasty and this reflected mild or no displacement.

**Figure 1:**
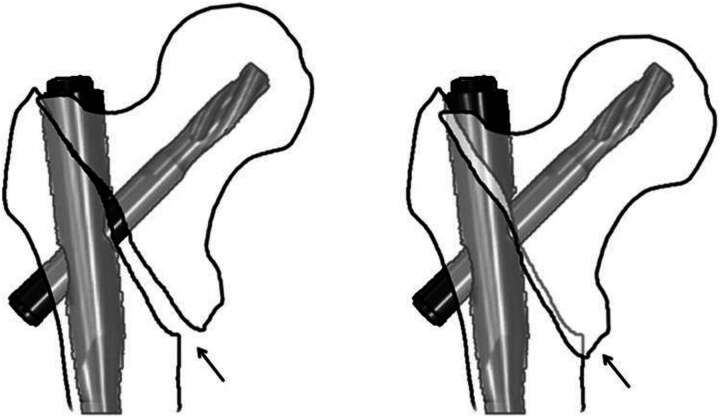
Positive medial cortical support prevents the medial head–neck fragment from impacting into the lateral shaft fragment. Arrows show contact between the cortexes. Reproduced from Chang et al.^8^

The quality of fracture reduction and metalwork placement were assessed using previously validated methods. Chang *et al* showed that nonanatomic reduction with positive medial cortical support (as well as anatomic reduction) in an unstable pertrochanteric hip fracture was a stable construct. It was concluded that a lateralised distal shaft fragment prevents the proximal medial neck fragment from impaction-displacement; therefore, medialised proximal fragment (Figure 1) enhances controlled fracture impaction with a sliding hip screw.^8^ We assessed the type of cortical contact between neck and shaft fragments in a pertrochanteric fracture on an anteroposterior (AP) view of the intraoperative fluoroscopy. Quality of reduction was classified into three categories: positive medial cortex support (74 cases), negative medial cortex support (18 cases) and neutral position (64 cases).

**Figure 2:**
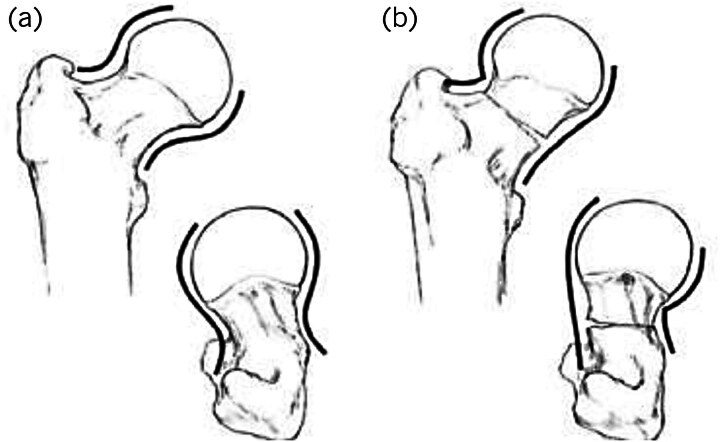
Lowell alignment theory states that satisfactory reduction will produce a “S” or “reverse S” in anteroposterior and lateral X-ray view (a), whereas malalignment will produce a “C” shape (b). Reproduced from Yoon HK, Dan JM. Femoral neck fracture. *J Korean Hip Soc* 2010; **22(1)**: 13–19. Copyright ^©^ 2010 The Korean Hip Society.

Lowell alignment theory was used to assess reduction of an intracapsular fracture except for the basicervical pattern (basicervical fractures were treated like the pertrochanteric fracture pattern). An S-shape or reverse S-shape reflects satisfactory reduction, in contrast to the C-shape on either the AP or lateral radiographs (Figure 2).^9^ A retrospective study conducted in Regensburg University Medical Center, Germany showed that the centrum–collum–diaphyseal (CCD) angle is one of the important factors in stability while reducing subtrochanteric fractures. They noted that the uncomplicated healing group has a higher CCD angle on the broken side than on the contralateral side.^10^

**Figure 3:**
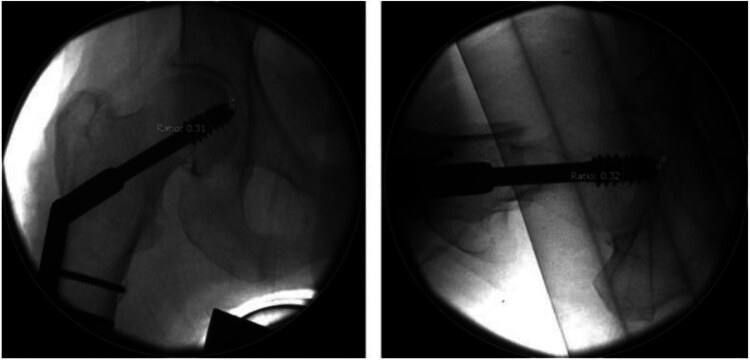
Wijeratna’s theory demonstrating that if the distance from the tip of the lag screw and the apex of the femoral head is < 12.5mm on both the fluoroscopic anteroposterior and lateral views, then the tip–apex distance will be <25mm. Reproduced from Wijeratna.^13^

**Figure 4:**
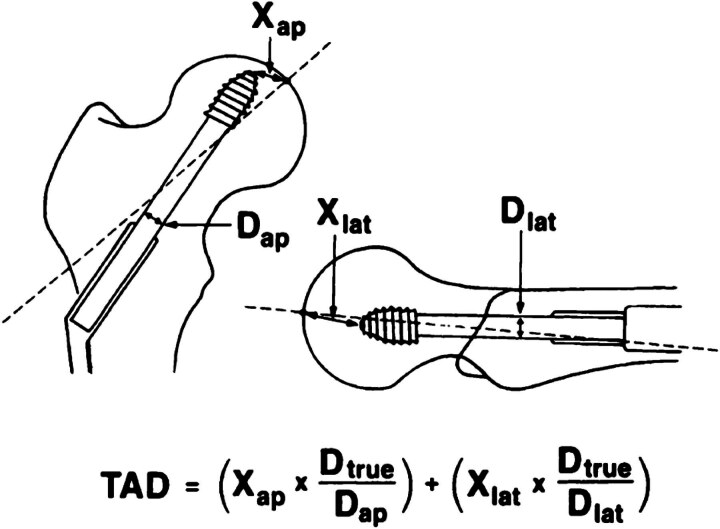
Drouinaud *et al*’s calculation of the tip–apex distance through intraoperative estimation. Reproduced from Drouinaud *et al*.^14^ Copyright ^©^ 2021 [Orthopaedics & Traumatology, Surgery & Research] Elsevier Masson SAS. All rights reserved.

The quality and consistency of screw placement in the femoral head in our cohort was analysed using the Baumgartner guidance for the tip–apex distance (TAD). Baumgartner *et al* recommended a TAD of <25mm to minimise risk of cut-out of the lag screw.^11,12^ Owing to the lack of postoperative images in most cases and different intraoperative magnifications, we looked at the intraoperative fluoroscopy as recommended by Wijeratna. The known thread diameter of the lag screw (12.5mm) for most of the screws used in hip fixation in the National Health Service was used as a standard reference. Wijeratna suggested that a distance between the tip of the lag screw and the apex of the femoral head of <12.5mm on both the fluoroscopic AP and lateral views will result in a TAD of <25mm.^13^ This eliminates the need to use the original formula of Baumgartner to calculate the TAD (Figure 3). The hypothesis was supported in a prospective study by Drouinaud *et al* in 2021 who showed that there was a high degree of concordance between intraoperative estimation of the TAD and its measurement using the formula (Figure 4) on postoperative images.^14^

It is reported that the inverted triangular configuration of cannulated compression screws for fixation of fractures of the femoral neck resulted in a lower incidence of screw cut-out. This was demonstrated in the finite element analysis evaluation by Li *et al*.^15^

### Statistical methods

Continuous variables were assessed visually for normality. DAP was non-normally distributed and log-transformed for subsequent analysis. Univariate linear regression analysis for associations between each independent variable and log-DAP was performed. A multivariate model for log-DAP was performed using statistically significant univariate factors (while maintaining independence) and surgeon level. Additional predetermined multivariate regression models (based on plausibility) were assessed for associations with log-DAP: (1) level of surgeon adjusting for complexity of fracture, fracture type and implant used; (2) quality of reduction and quality of fixation; and (3) complexity and quality of reduction and quality of fixation. Comparisons between categorical data were performed using the chi-squared test. The data were analysed listwise. The data are presented as mean ± SD. Analysis was performed using SPSS (version 25, IBM Corp, Armonk, NY, USA). A *p*-value of <0.05 was deemed statistically significant.

## Results

### Patient characteristics

Patient characteristics and surgical details are shown in Table 1. Some 184 patients were included, and DAP values were available for 177. The mean age was 83.5 years (±8.6) and 69% of patients were female.

**Table 1:** Patient and surgical characteristics and univariate regression models to assess determinants of dose–area product

Factor	Number (%) or mean ± SD (*n *= 184)	DAP for each subgroup (cGy/cm^2^)	*B* (SE)	Standardised Beta	*p*-value
Sex			0.004 (0.052)	0.006	0.938
Female	127 (69.0)	80.8 ± 117.4			
Male	57 (31.0)	70.6 ± 63.4			
Age (years)	83.5 ± 8.6	–	0.005 (0.003)	0.126	0.095
Fracture subtype			0.046 (0.014)	0.246	**0.001**
Simple pertrochanteric	51 (27.7)	54.5 ± 54.0			
Multi-fragmented PT	87 (47.3)	69.7 ± 54.8			
Reverse oblique	12 (6.5)	103.0 ± 67.9			
Basi-cervical	7 (3.8)	51.6 ± 50.3			
Trans-cervical	5 (2.7)	203.5 ± 241.6			
Subcapital	11 (6.0%)	36.0±12.6			
Subtrochanteric	11 (6.0%)	226.7±303.3			
Fracture complexity			0.173 (0.048)	0.265	**<0.001**
Simple	74 (40.2)	61.7 ± 84.0			
Complex	110 (59.8)	88.5 ± 113.8			
Implant type			0.060 (0.016)	0.277	**<0.001**
2-hole DHS	13 (7.1)	90.4 ± 164.5			
3-hole DHS	0	–			
4-hole DHS	87 (47.3)	57.2 ± 50.4			
5-hole DHS	9 (4.9)	94.0 ± 126.6			
Short PFN	37 (20.1)	74.3 ± 42.5			
Long PFN	35 (19.0)	125.7 ± 179.8			
3 cannulated screws	3 (1.6)	41.6 ± 25.7			
Additional implant			0.315 (0.094)	0.246	**0.001**
Yes	13 (7.1)	159.8 ± 174.1			
Not used	171 (92.9)	71.6 ± 94.3			
Reduction satisfactory			0.142 (0.078)	0.136	0.070
Yes	163 (88.6)	75.6 ± 105.3			
No	21 (11.4)	94.4 ± 85.8			
Fixation satisfactory			0.185 (0.059)	0.229	**0.002**
Yes	148 (80.4)	71.1 ± 105.5			
No	36 (19.6)	103.8 ± 90.8			
Operative time (min)	90.0 ± 27.4	–	0.005 (0.001)	0.459	**<0.001**
Surgeon level			0.053 (0.029)	0.137	0.069
Junior	14 (7.6)	44.2 ± 26.4			
Middle	62 (33.7)	68.3 ± 92.1			
Senior	80 (43.5)	86.5 ± 63.2			
Unknown	28 (15.2)	89.2 ± 200.9			

DAP = dose–area product; DHS = dynamic hip screw; PFN = proximal femoral nail; PT= pertrochanteric; DAP scores were log-transformed for analysis to correct normality

More than half of patients had a complex fracture pattern (59.8%). We categorised types 31A2 and 31A3 hip fractures as complex (110 in total). Simple pertrochanteric fractures (51 patients) in addition to intracapsular neck of femur fractures (23 cases) were considered a simple fracture pattern.

The most common implant used was a 4-hole DHS (47.3%), followed by short and long proximal femoral nail (PFN) (20.1% and 19.0%, respectively). An additional implant was required in 13 operations (derotational screws were used in eight cases with subcapital fracture neck of femur and cables used in five patients: two subtrochanteric, two multi-fragmentary pertrochanteric and one reverse oblique fracture).

Most operations were completed by senior surgeons (43.5%); middle-grade surgeons led 33.7% of operations and junior surgeons led 7.6%. The remaining 28 operations were led by a surgeon of unknown level. The average DAP exposure over the whole cohort was 77.6 ± 103.3 cGy/cm^2^.

### Univariate associations of DAP

Results of the univariate analysis are shown in Table 1. There were significant associations of fracture subtype (*p *= 0.001), fracture complexity (*p *< 0.001), implant type (*p *< 0.001), whether an additional implant was used (*p *= 0.001), whether fixation was satisfactory (*p *= 0.002) and operative time (*p *< 0.001) with DAP during surgery. There was some evidence of an association between the level of surgeon and DAP, although this was not statistically significant (*p *= 0.069).

Surgeon level was associated with different fracture complexity (χ^2^ = 9.55, *p *= 0.023), with junior surgeons completing a greater proportion of simple fractures (64.3%), than middle-grade surgeons (48.4%) and senior surgeons (28.7%). Likewise, the implants used by junior and middle-grade surgeons differed from those used by senior surgeons (χ^2^ = 39.6, *p *= 0.001).

### Multivariate model

In the multivariate regression model for association with DAP, significant predictors were use of an additional implant, implant type and whether fixation was satisfactory (Table 2).

**Table 2:** Multivariate regression model to assess for determinants of dose–area product

Factor	*B* (SE)	Standardised beta	*p*-value
Surgeon level	0.028 (0.028)	0.072	0.317
Additional implant	0.332 (0.088)	0.260	**<0.001**
Implant type	0.042 (0.017)	0.193	0.018
Fixation satisfactory	0.137 (0.056)	0.169	0.016
Fracture complexity	0.092 (0.052)	0.141	0.075
Reduction satisfactory	0.086 (0.072)	0.082	0.236

Dose–area product (DAP) scores were log-transformed for analysis to correct normality

#### Predetermined multivariate models

Multivariate regression assessing surgeon level with DAP found no significant association after adjusting for fracture complexity (*B* = 0.038, SE = 0.029, *p *= 0.180), fracture type (*B* = 0.041, SE = 0.029, *p *= 0.152) or implant used (*B* = 0.025, SE = 0.029, *p *= 0.390).

Multivariate regression assessing quality of fracture reduction and fixation with DAP found no change from univariate analysis of these variables. Similarly, a multivariate model assessing reduction and fixation satisfaction and fracture complexity with DAP found no change from univariate analysis.

## Discussion

On the one hand, the image intensifier is a vital tool used in orthopaedic trauma procedures. On the other hand, it carries some risks to patients and, to a greater extent, theatre staff. Internal fixation of hip fractures is one of the most common orthopaedic procedures performed in UK practice. Many studies have reviewed the effect of different factors affecting radiation exposure to encourage surgeons to pay attention to those that are modifiable. In the same context, we performed this study to review levels of radiation exposure in our trust and analyse some of the contributing factors when performing one of the most common procedures in the UK.

One of the recommendations of “The Ionising Radiation (Medical Exposure) Regulations 2000”, is that “the employer should set diagnostic reference levels” for “radiological procedures” in quantities that can be measured, such as DAP and/or screening time.^16^ Therefore, these parameters were used to quantify the dose of radiation exposure during hip fracture surgery. We retrospectively reviewed a cohort of patients with hip fractures who underwent surgical fixation over a period of 12 months. We analysed the DAP and dS as recorded on the radiology report of the intraoperative images; only seven cases (3.8%) did not have the report attached. This number of missing cases is considered negligible in comparison with the total number of cases reviewed.

This study analysed different factors (surgeon level of experience, complexity of the fracture and implants used) that influence intraoperative radiation exposure. In previous literature, the most discussed factors were surgeon level of experience, the fracture pattern, the implant used and, to a lesser extent, the experience of the radiographer and the position of the patient. In four studies, it was pointed out that a higher dose of radiation was recorded when junior surgeons were the primary operators.^16–19^ Although Giannoudis *et al* reported a higher dose of radiation when the operating surgeon was junior, they also confirmed that more complex fractures (four-part fracture of the proximal femur) required a higher radiation dose and longer screening time.^20^ In the same context, Baratz *et al* found that the strongest determinants of radiation dose were the fracture pattern, patient body position, patient body mass index (BMI) and the use of cephalomedullary devices.^21^

The method for classifying surgeon grade differed between the quoted studies. For example, Botchu *et al* classified operating surgeons into two categories only, which represent the two extremes of the surgical experience.^16^ However, we classified the cohort of operating surgeons into three groups, as did Bruce *et al* and Rashid *et al*.^19,22^ Bruce *et al* identified the consultant as someone who is fully qualified with a certificate of training who can practise independently, whereas senior and junior trainees are surgeons who have >5 years and <5 years of experience in trauma and orthopaedics, respectively. Rashid *et al* compared the radiation exposure between consultants, staff grade and associate specialist doctors, and specialist training registrars. This method can be criticised because this classification does not accurately reflect the years of experience of the operating surgeon. In our study, we classified surgeons into three categories, and we believe that this method stratifies more accurately the level of experience of the surgeons. The only other study that subdivided surgeons into more detailed categories was that of Quah *et al*.^18^ They classified operating surgeons into five groups, which gave good stratification of the surgeons. Although fixation of hip fracture is assigned most of the time to surgeons below the consultant level, junior and middle-grade surgeons in our study were the first operators in 14 and 62 operations, respectively. Both groups performed 76 of 184 procedures (41.3%). However, Bruce *et al* in 2021 reported 83.9% (225 of 268 procedures) of hip fixation procedures were done by non-consultant trainees.^22^ In our study, inaccurate documentation is thought to be the reason for the lower number of reported non-consultant-led procedures.

We subdivided the hip fractures and correlated the complexity of these subtypes and the implant used with the amount of radiation exposure. We reported that fracture complexity led to greater operative time and fluoroscopic radiation (DAP was 88.5 ± 113.8cGy/cm^2^ for complex fractures in comparison to 61.7 ± 84.0cGy/cm^2^ in simple fractures). Bruce *et al* in their study published in 2021 also analysed the effect of fracture complexity and level of training of the operating surgeons on the radiation dose delivered. The study results were like our conclusion in that higher DAP values were recorded with more complex fractures. However, Bruce *et al* confirmed the inverse relationship between the grade of operating surgeon and the radiation dose.^22^ We reviewed all hip fracture subtypes including the subtrochanteric fracture pattern (11 hips of 184; 6% of the included cohort). To our knowledge, this fracture subtype was excluded from the study population in all the quoted studies except Buxbaum *et al*.^23^ Average DAP exposure during fixation of subtrochanteric fractures in our cohort was 226.7 ± 303.3cGy/cm^2^, which is 148.4cGy/cm^2^ more than the average DAP (77.6 ± 103.3 cGy/cm^2^) for the whole cohort of patients. In Buxbaum *et al*’s study, they reported the longest fluoroscopy times during subtrochanteric fracture fixation (176.1 ± 11.27s) among all the other fracture patterns, but they did not report the DAP values.^23^

The type of implant used in fixation of a hip fracture affects the amount of radiation exposure. We confirmed higher DAP values with the use of PFN in comparison with the DHS in proximal femur fixation, as reported by Rashid *et al* and Kelly *et al*.^19,24^ Additional implants like a derotational screw or cerclage wire needed more operative time for insertion and subsequently greater radiation exposure (159.8 ± 174.1cGy/cm^2^). The prolonged operative time for the insertion of additional implants applies to the eight cases of the intracapsular fracture neck of femur where derotational screws were used. However, the five complex cases (two subtrochanteric, two multi-fragmentary pertrochanteric and one reverse oblique fracture) which were fixed with cerclage wires and PFN were primarily associated with high radiation exposure because of the fracture complexity.

To our knowledge, this study is the first to correlate the quality of reduction and implant placement in fixation of neck of femur fractures with the amount of intraoperative radiation. Validated methods were used to assess the quality of reduction and implant placement. We reviewed many factors that can affect the amount of intraoperative radiation during hip fracture fixation. We analysed all possible hip fracture patterns including the subtrochanteric fracture pattern. This pattern was not included in many studies with similar objectives.^16,17,22^

### Study limitations

One of the weaknesses in our study is the relatively small cohort of patients, although this could still be considered a reasonably sized cohort of hip fractures compared with other series. Although Buxbaum *et al* reviewed 852 extracapsular proximal femur fractures, they only correlated the fluoroscopy screening time with the level of the surgeon, fracture type and implant used.^23^ Their results were like ours regarding the correlation between fracture pattern, implant used and radiation exposure, but were in contrast to our observation on the effect of the seniority of the operating surgeon on radiation exposure. Although Buxbaum *et al* used only fluoroscopy screening time as an indicator of radiation exposure, we believe that there is a direct relationship between screening time and DAP values.

We could not report the effect on radiation exposure for some factors analysed in other studies (e.g. patient BMI, body position and the experience of the radiographers). We could not analyse the above-mentioned factors because of the retrospective nature of our study and incomplete documentation. We could not review the postoperative images of our cohort to assess the effect of quality of reduction and fixation on the long-term outcome because we do not routinely arrange long-term follow-up for fracture neck of femur patients in our trust. However, assessment of functional outcome was beyond the goals of our study.

On reviewing the operative records during the data collection stage, we noted that there were some missing data. We could not retrieve the radiation report in seven cases. In addition, there were 28 cases in which the primary surgeons were not documented properly. We believe that one of the reasons for this is that some of the reviewed data were obtained from an archived source. We obtained the operative records from two different systems (ORMIS and Bluespier). The former source was all archived by the time of the data collection.

### Basic principles and personal protective equipment

**Figure 5:**
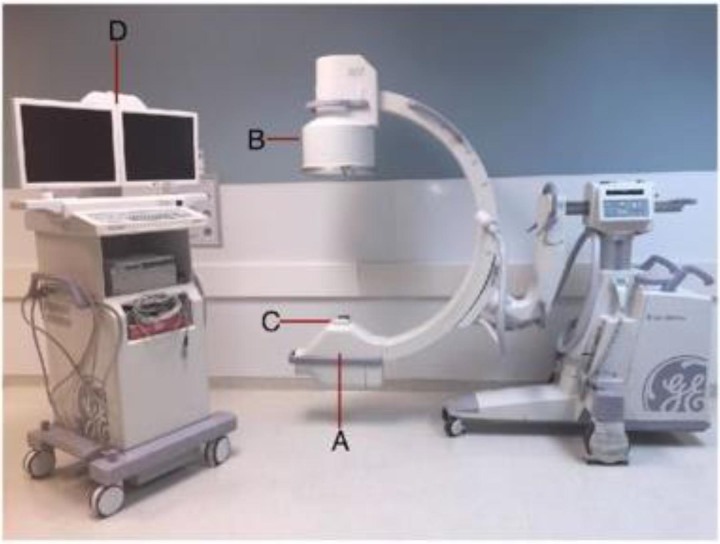
Basic C-arm unit: (a) X-ray tube; (b) image intensifier; (c) collimator; and (d) display monitor. Reproduced from Kaplan *et al*.^26^

Understanding basic principles behind the use of fluoroscopy in operating theatres is important to minimise exposure to ionising radiation. It was noted in the Radiation In Orthopaedics (RIO) study that there is lack of training in radiation safety among orthopaedic surgeons in the UK.^25^ An image intensifier (C-arm machine) consists of four main parts (the X-ray tube produces the beam, the collimator controls shape of the beam, the image intensifier receives the beam and transforms into an image, and the display monitor shows the image) (Figure 5).^26^ C-arm position is crucial to minimise the amount of scattered radiation. The deflected radiation or the scatter travels with less energy but accounts for most of the effect of radiation exposure on theatre staff.^27^ Therefore, adequate orientation of the C-arm reduces the effect of scatter.^26^

Wearing appropriate well-fitted radiation protection equipment is mandatory in theatres. This includes a lead apron, thyroid shield, lead protective glasses and gloves, in addition to a radiation dosimeter or badge. Some of this equipment is not accessible to theatre staff in many workplaces, and this may be due to a lack of awareness of their significance. This was observed in the RIO study (where >50% of respondents stated that they used thyroid shields, lead protective gloves and glasses, and dosimeters only sometimes, rarely, or never).^25^

The current risk of breast cancer is not known in orthopaedic surgeons. However, in three different studies conducted in the US, Chou *et al* showed a 1.9-fold increase in all-cause cancer incidence and a 2.9–3.9-fold increase in breast cancer incidence among orthopaedic surgeons. Lack of a similar observation among plastic surgeons and urologists may or may not suggest a correlation between increased cancer risk and radiation exposure.^28–30^ Because standard tabard-style gowns do not protect the upper outer quadrant of the breast,^31^ other options are available for UK practice. These options were published in the *Journal of Trauma and Orthopaedics* and adopted by the British Orthopaedic Association in June 2023.^32^ In this article, Sevenoaks and her working group advised the use of well-fitted vests with high cover to the axilla instead of the standard tabard. Also, standing away from the source, keeping arms by the side, standing square to the source, and reducing the use of direct lateral views can minimise breast irradiation. New garment types which cover the breast tissue are under different stages of development by manufacturers.^32^

## Conclusion

Increased radiation in proximal femur fractures is seen in the fixation of complex fractures, certain fracture subtypes, the type of implant used and the use of an additional implant. Surgeon seniority did not result in a lower radiation exposure, which is in contrast with other published studies. Surgeons are advised to learn the hazards of radiation exposure and factors affecting it. Senior surgeons are responsible for teaching more junior doctors how to minimise intraoperation radiation exposure.
